# Clinical Value of Plasma Soluble Urokinase-Type Plasminogen Activator Receptor Levels in Term Neonates with Infection or Sepsis: A Prospective Study

**DOI:** 10.1155/2014/375702

**Published:** 2014-05-05

**Authors:** Tania Siahanidou, Alexandra Margeli, Chrysanthi Tsirogianni, Stavroula Charoni, Maria Giannaki, Eustathios Vavourakis, Athina Charisiadou, Ioannis Papassotiriou

**Affiliations:** ^1^Neonatal Unit, First Department of Pediatrics, Athens University Medical School, “Aghia Sophia” Children's Hospital, 115 27 Athens, Greece; ^2^Department of Clinical Biochemistry, “Aghia Sophia” Children's Hospital, 115 27 Athens, Greece; ^3^Department of Microbiology, “Aghia Sophia” Children's Hospital, 115 27 Athens, Greece; ^4^Hematology Laboratory, “Aghia Sophia” Children's Hospital, 115 27 Athens, Greece

## Abstract

*Background*. suPAR, the soluble form of the urokinase-type plasminogen activator receptor, has been identified as a biomarker of infection in adults but its properties in neonatal infection are not known. *Methods*. Plasma suPAR levels were determined by ELISA in 47 term neonates with infection (19 bacterial and 28 viral) and in 18 healthy neonates as controls. Thirteen out of 47 infected neonates were septic. In all infected neonates, suPAR levels were repeated at 24 hours, 48 hours, 3–5 days, and 7–10 days following admission. *Results*. Plasma suPAR levels were significantly increased in infected neonates upon admission, whereas they were highest in septic neonates, in comparison with controls (*P* < 0.001) and correlated positively with serum CRP levels (*P* = 0.001). At infection subsidence, suPAR concentrations decreased significantly in comparison with baseline (*P* < 0.001) but remained higher than in controls (*P* = 0.01). Receiver operating characteristic analysis resulted in significant areas under the curve for detecting either infected or septic neonates, but not for discriminating between bacterial and viral cause of infection. *Conclusions*. suPAR is a diagnostic biomarker of infection or sepsis in term neonates; however, it cannot discriminate bacterial from viral infections and also its utility for monitoring the response to treatment is questioned.

## 1. Introduction

It is well known that early diagnosis and management of neonatal infection is mandatory for outcome [1]. Clinical difficulties in identifying early the infection in neonates and also in discriminating between bacterial and viral aetiology have led to the evaluation of several biomarkers including hematologic parameters, acute phase proteins, chemokines, cytokines, and cell-surface antigens [1, 2]. However, none of the biomarkers of neonatal infection or sepsis assessed up to date has been shown to be ideal [2].

The urokinase-type plasminogen activator receptor (uPAR), a membrane-linked receptor with extracellular protease activity that transduces intracellular signaling pathways, is expressed on the surface of several inflammatory cells, including neutrophils, lymphocytes, and monocytes, as well as on endothelial cells [3]. It is the cognate receptor for urokinase-type plasminogen activator which is activated upon binding and involved in the conversion of plasminogen to the active fibrinolytic enzyme plasmin [3]. After cleavage and release from the cell membrane, uPAR is recognized as a soluble receptor (suPAR). Increased suPAR levels have been reported in several biological fluids including blood, urine, and saliva, as well as in cerebrospinal, pleural, pericardial, and peritoneal fluid [3–5], in response to a variety of infections in adults [6–8], and they correlate positively with the activation level of the immune system [9]. In neonates, the properties of suPAR as a biomarker of infection are not known; to the best of our knowledge, the only information regarding suPAR in neonatal infections derives from one study aimed to identify by multiplexed immunoassay arrays differentially expressed serum proteins in clinically infected and noninfected preterm infants [10].

The objective of the present study was to evaluate the clinical value of suPAR in the detection of neonatal infection or sepsis, discrimination between bacterial and viral infections, and monitoring the responsiveness to treatment.

## 2. Materials and Methods

### 2.1. Subjects and Study Protocol

Criteria for eligibility in the study included: (1) term infant, (2) postnatal age between 4 and 28 days of life, (3) bacterial (confirmed by positive blood, urine, and/or CSF culture) or viral infection (confirmed by detection of virus in biological fluids or highly probable viral infection suggested by accompanying symptoms, well-appearing, negative bacterial cultures, normal for age total leukocyte count/differentiation, and serum C-reactive protein [CRP] values less than 10 mg/L). Exclusion criteria were as follows: (1) perinatal asphyxia, (2) antibiotic treatment in the last 10 days before admission, (3) surgery, and (4) positive culture(s) for* Staphylococcus epidermidis*.

On admission to our Special Care Unit, all infected neonates underwent blood, CSF, and suprapubic urine sampling for analyses and cultures; a small quantity of plasma (0.2 mL) was isolated within 30 min and stored at −80°C for measurement of suPAR levels. Additional biological specimens including synovial fluid, stools, throat swabs, or nasopharyngeal secretions were also obtained if required according to the presenting symptoms. Following admission, blood samples were drawn from all infected infants at 24 hours, 48 hours, 3–5 days, and 7–10 days for routine blood tests (full blood count, renal and liver function, and serum CRP levels), as well as for 0.2 mL plasma isolation and storing at −80°C for suPAR levels determination.

Plasma suPAR concentrations were finally measured in 47 infected neonates of mean ± SD gestational age 39.0 ± 1.0 weeks, birth weight 3, 135 ± 351 g, postnatal age 18 ± 6 days, and male/female ratio 29/18, and in 18 healthy term infants who had similar gestational age (38.6 ± 1.0 weeks), birth weight (3, 216 ± 406 g), postnatal age (17 ± 6 days), and gender distribution (11 males, 7 females) to those of infected infants, as controls. Five serial suPAR measurements were performed in each patient during the course of the disease, whereas suPAR plasma levels were determined only once in the control group along with full blood count, renal and liver function tests, and serum CRP levels. In all infants studied, associations of suPAR concentrations on admission with clinical and laboratory parameters (leukocyte count and differentiation, platelet count, serum CRP levels, renal and liver function) were assessed.

Nineteen out of 47 infected neonates suffered from bacterial infections confirmed only by blood (*n* = 4), urine (*n* = 8), or CSF (*n* = 1) cultures, or by both blood/urine (*n* = 4), blood/CSF (*n* = 1), and blood/synovial fluid (*n* = 1) cultures. The remaining 28 neonates had definite (*n* = 12) or probable (*n* = 16) viral infections (Table 1) with no evidence of bacterial super-infection. The definite diagnosis of viral infections was performed by polymerase chain reaction (enterovirus, adenovirus), immunochromatographic tests (adenovirus or Rotavirus in faeces) or direct immunofluorence in nasopharyngeal secretions (respiratory syncytial virus). Thirteen out of 47 infected neonates were septic. Sepsis was defined according to the criteria established by the international pediatric sepsis consensus conference [11]; evidence of systemic inflammatory response syndrome (SIRS) in the presence of or as a result of suspected or proven infection was required [11].

The Children's Hospital Ethics Committee approved the study and informed parental consent was also obtained.

### 2.2. Assays

Hematologic parameters were measured using a Siemens-ADVIA 120 whole blood autoanalyzer (Siemens Healthcare Diagnostics, Tarrytown, NY, USA). Blood Chemistry for renal and liver function was performed using the Siemens Advia 1800 Clinical Chemistry System (Siemens Healthcare Diagnostics, Tarrytown, NY, USA), whereas CRP concentrations were determined with immunonephelometry using a Beckman Coulter IMMAGE immunochemistry system (Beckman Coulter, Inc., Fullerton, CA, USA). The intra- and interassay coefficients of variation (CVs) were 3.5% and 7.0%, respectively.

For suPAR determination, plasma was isolated from EDTA-K_3_ anticoagulated blood samples of neonates following centrifugation at 3000 g for 10 minutes and stored at −80°C until analysis. Plasma suPAR levels were determined using a commercial enzyme-linked immunosorbent assay (ELISA) (suPARnostic Standard kit; ViroGates A/S, Birkerød, Denmark). This assay utilizes a double monoclonal antibody that measures all circulating suPAR, including full-length and cleaved forms of the receptor. According to the standards provided by the manufacturer in ng/mL, a curve was constructed by us and the results were expressed as ng/mL, in a range between 0.6 and 20.0 ng/mL. The intra- and interassay CVs ranged from 1.3% to 4.7% and 1.7% to 5.1%, respectively, whereas the sensitivity limit was 0.1 ng/mL.

### 2.3. Statistical Analyses

The sample size of the study was calculated using our preliminary results of plasma suPAR concentrations in infected and healthy neonates. Assuming an alpha risk of 0.05, a power of 0.80, and a bilateral test, it was estimated that 15 neonates was the minimum number of infants needed in each group to detect a significant difference of one SD in mean suPAR levels between infected and healthy infants.

Data are presented as mean ± SD, unless otherwise noted. Values of suPAR were not normally distributed overall (Kolmogorov-Smirnov test), but the distribution was normal for neonates with infections (bacterial or viral) and controls separately. Comparisons of quantitative variables between more than two groups were conducted by ANOVA with Bonferroni post hoc analysis or by Kruskal-Wallis and Mann-Whitney *U* test, as appropriate. A one-way repeated measures ANOVA was used to compare the values of suPAR with baseline (on admission) within the infected group, in bacterial and viral infections separately. Correlation (Pearson's or Spearman's correlation test, as appropriate) and regression analyses were used to examine relations among the variables of interest. The diagnostic properties of suPAR concentrations on admission were assessed by receiver operating characteristic (ROC) analysis.

Levels of statistical significance were set at *P* ≤ 0.05. All statistical analyses were performed using the SPSS statistical package (SPSS, version 19.0, Chicago, IL).

## 3. Results

### 3.1. On Admission to the Unit

Demographic, clinical characteristics, and laboratory findings including white blood cells (WBC), absolute neutrophil count (ANC), immature neutrophil and platelet count, liver and renal biochemistry, serum CRP, and plasma suPAR levels in infants with bacterial and viral infections, separately, on admission to the Unit, as well as in controls, are shown in Table 2. Infants with bacterial infections did not differ significantly than infants with viral infections in gestational and postnatal age, birth weight, gender, duration of fever before admission, and maximum body temperature (Table 2). The time for fever recession following admission was less than 36 hours in equal percentage (approximately 75%) of infants in the bacterial and viral infection group, but it was more than 72 hours in 3 out of 28 infants with viral infections (10.7%) and in none infants with bacterial infection; however, the difference between groups was not statistically significant (Table 2). As expected, the WBC, ANC, and serum CRP values were significantly higher in neonates with bacterial infections than those with viral infections and controls; immature neutrophil count was also higher, whereas platelet count was lower in neonates with bacterial infections but the difference between groups was not significant (Table 2).

Baseline mean ± S.D. plasma suPAR levels were significantly higher (*P* < 0.001) in the entire group of infected neonates (5.01 ± 1.52 ng/mL), as well as in neonates with bacterial or viral infections separately (*P* < 0.001 and *P* = 0.02, resp.) in comparison with controls (3.61 ± 0.74 ng/mL). No difference was recorded in suPAR levels between neonates with bacterial infections and those with viral infections (Table 2). Furthermore, in the group of neonates with viral infections, suPAR levels did not differ significantly between neonates with confirmed and those with probable infections.

**Figure 1:**
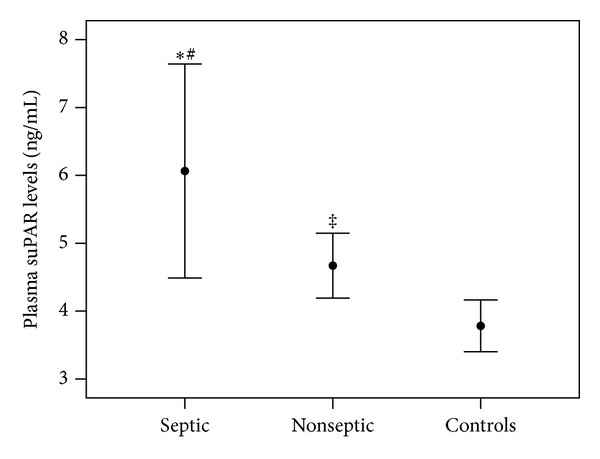
Plasma suPAR levels in septic, and nonseptic neonates, and controls. The bars represent mean (95% confidence interval for mean) of suPAR concentrations on admission. *P ≤ 0.001, ^‡^ *P* ≤ 0.05 compared with controls; ^#^ *P* < 0.05 compared with nonseptic neonates.

In the 13 out of 47 infected neonates who fulfilled the criteria for sepsis (11 with bacterial and 2 with viral infections), baseline mean ± S.D. plasma suPAR levels (6.05 ± 1.96 ng/mL) were significantly higher than levels in the remaining 34 nonseptic neonates (4.58 ± 1.08 ng/mL; *P* = 0.002) and controls (*P* < 0.001). The difference in suPAR levels between nonseptic neonates and controls was also significant (*P* = 0.02) (Figure 1).

**Figure 2:**
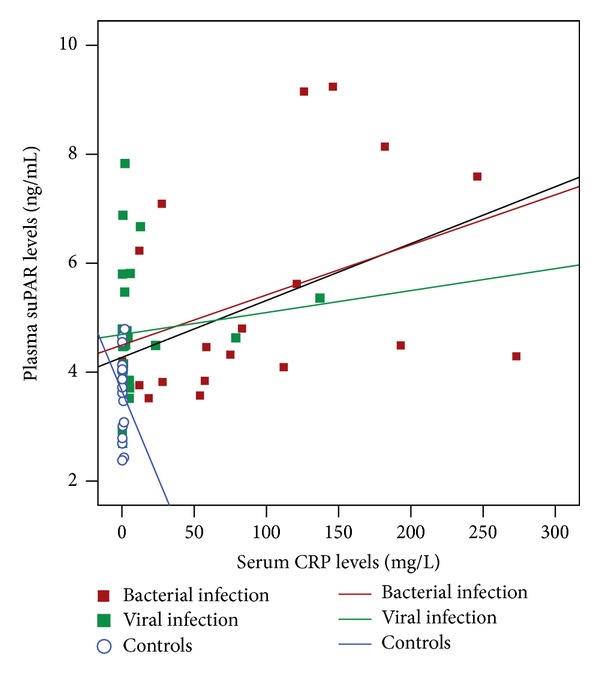
Correlations between plasma suPAR and serum CRP levels on admission. Red box, neonates with bacterial infections; green box, neonates with viral infections; blue circle, controls. The lines represent the regression slope separately for the groups of bacterial (red line; *P* = 0.02), viral infection (green line; *P* = 0.45), controls (blue line; *P* = 0.48), and for the entire study population (—; *P* = 0.001).

In the entire study population, suPAR levels on admission correlated positively with postnatal age (*r*_*s*_ = 0.29, *P* = 0.01), WBC (*r*_*s*_ = 0.26, *P* = 0.03), serum urea (*r*_*s*_ = 0.28, *P* = 0.03), and CRP levels (*r*_*s*_ = 0.42, *P* = 0.001). The positive correlation between suPAR and CRP concentrations was driven by the group of neonates with bacterial infections (*r*_*s*_ = 0.52, *P* = 0.02) (Figure 2). Moreover, suPAR levels correlated negatively with platelet number in neonates with bacterial infections (*r* = −0.55, *P* = 0.01), whereas a positive influence of male gender on suPAR levels was recorded in controls (regression coefficient *β* = 0.516, *P* = 0.02).

### 3.2. Diagnostic Value of suPAR Levels on Admission

**Figure 3:**
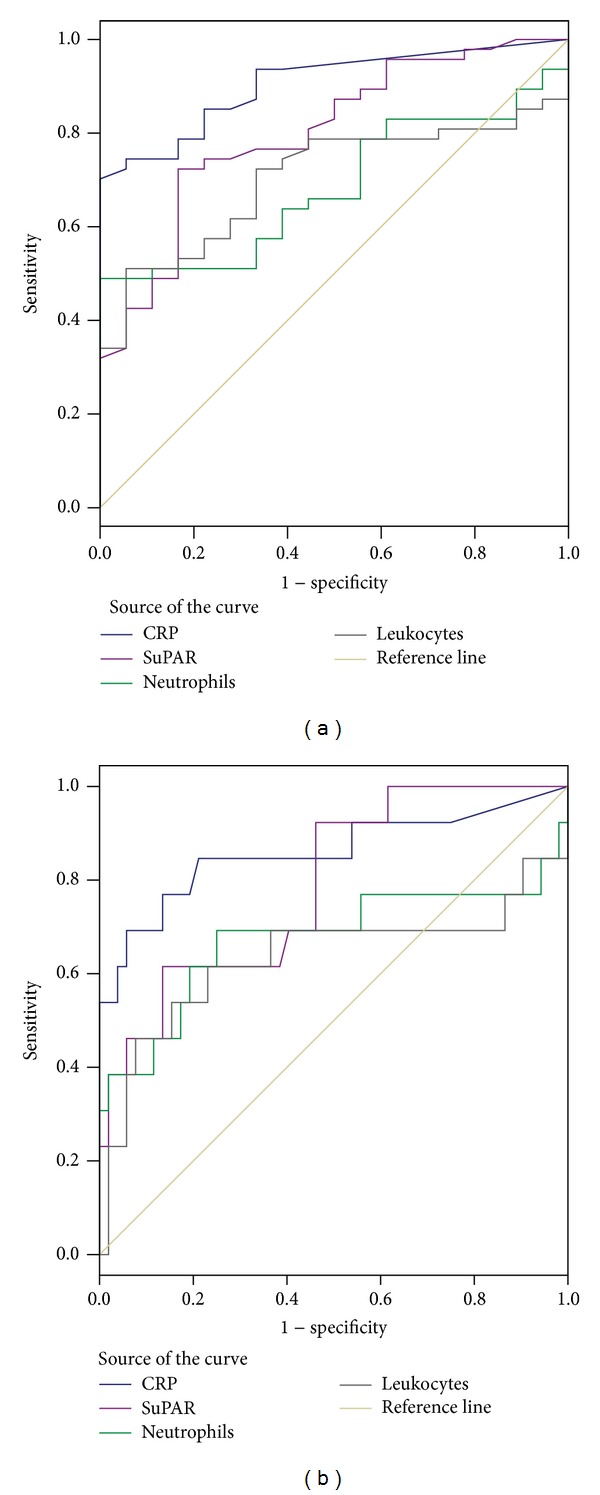
ROC curves for suPAR, CRP, WBC count, and ANC on admission for prediction of infection (Figure 3(a)) or sepsis (Figure 3(b)).

Receiver operating characteristic (ROC) analysis of suPAR values on admission to the Unit resulted in significant areas under the curve (AUC) for detecting either infected (AUC = 0.801 (95% CI: 0.687–0.916), *P* < 0.001) or septic neonates (AUC = 0.788 (95% CI: 0.653–0.923), *P* = 0.001) (Figures 3(a) and 3(b), resp.). However, the diagnostic performance of suPAR was not superior compared to CRP either for detecting infection (AUC = 0.905 (95% CI: 0.834–0.976), *P* < 0.001) or sepsis (AUC = 0.858 (95% CI: 0.714–1.000), *P* < 0.001), whereas it was almost equal compared to WBC and ANC for detecting infection (AUC = 0.787 (95% CI: 0.634–0.940), *P* = 0.001, and AUC = 0.820 (95% CI: 0.676–0.963), *P* < 0.001, resp.), but better than WBC and ANC for detecting sepsis (AUC = 0.633 (95% CI: 0.413–0.853), *P* = 0.14, and AUC = 0.675 (95% CI: 0.461–0.888), *P* = 0.05, resp., Figure 3). In our study population, SuPAR values higher than 4.07 ng/mL on admission could detect infection with sensitivity 74.5% and specificity 78%, whereas values greater than 4.79 ng/mL could detect sepsis with sensitivity 61.5% but specificity 89%. However, SuPAR values on admission could not discriminate between bacterial and viral cause of infection (AUC = 0.515 (95% CI: 0.331–0.699), *P* = 0.86).

### 3.3. Serial Measurements of suPAR in Neonates with Infection

Within the entire group of infected neonates, the time points at which repeat blood samples were obtained had a significant effect on plasma suPAR levels (*F* = 10.5, *P* < 0.001) by one-way repeated measures ANOVA, whereas the effect of the infection type (bacterial or viral) was not significant.

**Figure 4:**
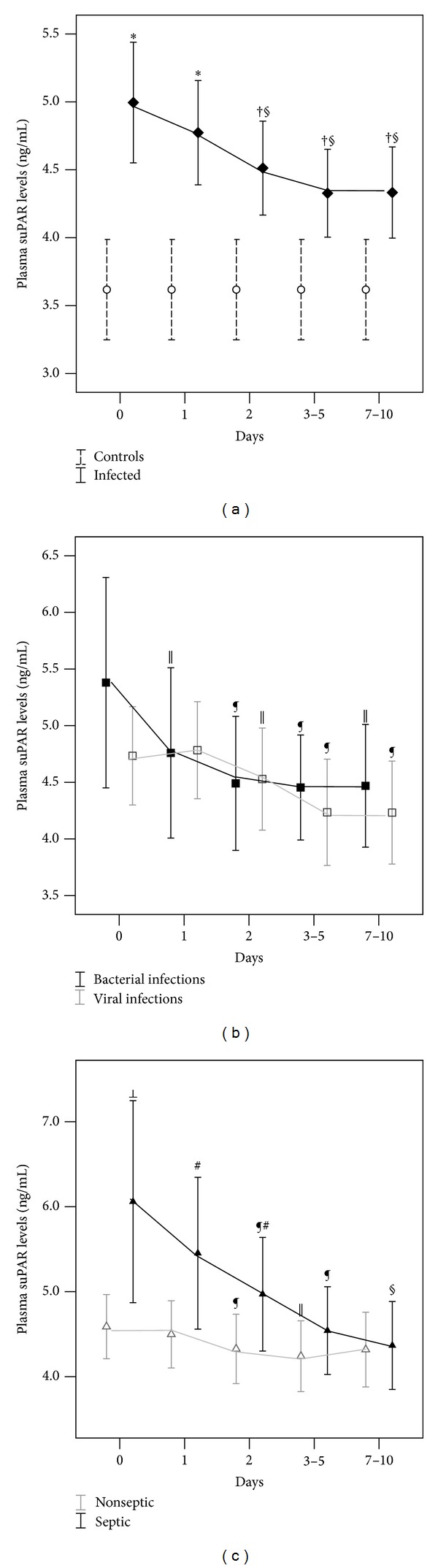
Repeated measurements of plasma suPAR levels during the course of infection. The bars represent mean (95% confidence interval for mean) of suPAR concentrations. (a) Decline in suPAR levels in the entire population of infected neonates (*P* < 0.001) and comparison with levels in controls at sequential time points. (b) Decline in suPAR levels separately for the groups of bacterial (*P* = 0.004) and viral infections (*P* < 0.001). (c) Decline in suPAR levels separately for the groups of septic (*P* < 0.001) and nonseptic (*P* = 0.016) infected neonates. *P ≤ 0.001, ^†^ *P* ≤ 0.01, compared with controls; ^§^ *P* ≤ 0.001, ^¶^ *P* ≤ 0.01, ^||^ *P* ≤ 0.05, compared with baseline; ^⊥^ *P* ≤ 0.01, ^#^ *P* ≤ 0.05, compared with nonseptic neonates.

In comparison with values on admission (baseline), suPAR concentrations decreased significantly (*P* < 0.001) in the entire population of infants with bacterial and viral infections (Figure 4(a)); however, at 7–10 days following admission, suPAR levels were still significantly higher in comparison with controls (*P* = 0.01) (Figure 4(a)). Plasma suPAR levels also declined significantly during the course of the disease in the group of neonates with bacterial (*P* = 0.004) and viral infections (*P* < 0.001), separately (Figure 4(b)), whereas no difference was recorded between the two groups. In neonates with bacterial infections, suPAR concentrations decreased significantly from baseline to 24 hours (*P* = 0.01), as well as from 24 to 48 hours (*P* = 0.04), whereas the alteration in suPAR levels from 48 hours to 3–5 days or from 3–5 days to 7–10 days following admission was not statistically significant. In neonates with viral infections, a significant decrease in suPAR levels was firstly observed between 24 and 48 hours (*P* = 0.007); suPAR levels also decreased from 48 hours to 3–5 days (*P* = 0.05), but they did not alter significantly from 3–5 days to 7–10 days following admission (Figure 4(b)).

In the septic group of infants, separately, suPAR concentrations decreased significantly from 24 hours onwards in comparison with baseline (*P* < 0.001) (Figure 4(c)), but remained higher than in controls at 7–10 days following admission (*P* = 0.05).

## 4. Discussion

According to the results of this study, circulating suPAR levels are increased in term neonates with acute infections in comparison with healthy controls. Moreover, suPAR levels in septic neonates are higher than levels in infected neonates who do not fulfill sepsis criteria. Increased systemic suPAR levels were previously reported in critically ill adults with or without SIRS, or sepsis [8]; a gradual increase in levels of suPAR was shown from adults who did not fulfill SIRS criteria to patients with SIRS and patients with sepsis [8, 9]. SuPAR levels were almost two times higher in septic adults than levels in our septic neonates, whereas levels in healthy adults were similar to those in our control group [8]. The difference in suPAR levels between septic neonates and septic adults can possibly be attributed to the less severity of sepsis in our neonates; all studied infants were hospitalized in a special, but not intensive, care unit and in none of them the course of disease was complicated or fulminant. suPAR levels in our study population were similar to those in older children with acute malaria infection [12], community-acquired pneumonia [13], and urinary tract infection [14]. There is only one published study of suPAR levels in neonates (10); suPAR has been recognized as one among eight proteins increased in clinically infected preterm infants, but neither its association with bacterial or viral cause of infection, or with the course of disease, was studied [10].

Despite the increased circulating suPAR levels, the diagnostic value of suPAR for identifying infection or sepsis in adults is questioned [9] as there are reports showing that it is good, uncertain, or poor [7, 8, 15, 16]. It was previously shown that in comparison with other, frequently used in clinical practice, biomarkers of sepsis including CRP and procalcitonin, suPAR was not a better diagnostic marker [7, 15]. In our study, the diagnostic value of suPAR for neonatal infection was good, whereas it was much better for neonatal sepsis. However, similarly to studies in adults [7, 16], suPAR was less accurate than CRP as diagnostic biomarker and also did not have any value in discriminating bacterial from viral infections. Furthermore, in neonates with bacterial infections, suPAR levels did not seem to correlate with bacteria species (data not shown).

Whereas the diagnostic utility of suPAR is controversial, several studies have shown that suPAR is a valuable prognostic biomarker [6, 16–19]. Circulating suPAR levels, but not CRP levels [20, 21], were reported to be higher on admission in nonsurvivor adults compared to patients who survived sepsis [6, 16–18]. The prognostic value of suPAR for mortality could not be evaluated in our study as all septic neonates survived. However, the higher suPAR levels on admission in septic neonates compared to infected, but nonseptic, ones and the negative correlation between suPAR levels and platelet number in the bacterial infant group indicate that suPAR is indeed associated with the severity of infection.

The positive correlation between suPAR and CRP levels in our study population is in accordance with the fact that circulating suPAR levels reflect the degree of immune activation and systemic inflammation [3]. There is also some evidence that individual factors, such as age and possibly female gender, are positively associated with systemic suPAR levels [9, 13, 22, 23]. In our study, suPAR levels correlated positively with postnatal age, whereas a positive influence of male, but not female, gender was observed in controls.

We wonder whether suPAR could be used as a biomarker of clinical recovery or response to treatment in patients with infection or sepsis. Changes in suPAR levels after initiation of antimicrobial therapy have been studied only in adults [8, 16, 19, 24]; it was shown that suPAR may remain elevated for days (even for weeks) after initiation of treatment. In our study, suPAR levels declined significantly in infected neonates; however, 7 to 10 days following admission, although all infected neonates had fully recovered, suPAR levels were higher than in healthy controls. Given that suPAR levels were not halved even at 7 to 10 days following recovery, we assume that either the half-life of suPAR, being unknown so far, is long or the production/release of suPAR is continued for a period of time following clinical improvement.

SuPAR participates in a number of immunological functions, including cell adhesion, migration, chemotaxis, proteolysis, immune activation, tissue remodeling, invasion, and signal transduction [3]. However, its precise role in infection and sepsis is still not clear. Knockout mice lacking uPAR have shown impaired host defense against sepsis, including reduced neutrophil migration towards the primary site of infection and markedly impaired phagocytosis in comparison with wild-type mice [25, 26]. On the other hand, uPAR and circulating suPAR have been implicated in the promotion of disease progression [25, 27]. Further studies are needed to elucidate whether suPAR is merely an epiphenomenon of the increased activation level of the immune system, or either this receptor possesses a protective role in neonatal sepsis or promotes inflammation/tissue damage and should, therefore, become a potential therapeutic agent or target in the future.

## Figures and Tables

**Table 1:** Causes of infection in infants studied.

	Biological samples positive for virulence factors			
	Blood	Urine	CSF	Other
Bacterial infections (*N* = 19)				
*E. coli* (*n* = 14)	7	10	1	
*Klebsiella sp*. (*n* = 1)	1	1		
*Enterococcus faecalis* (*n* = 1)		1		
*Staphylococcus aureous* (*n = *2)	2			Synovial fluid (1)
*Streptococcus* group B (*n = *1)			1	
Viral infections (*N* = 28)				
Adenovirus (*n =* 3)				Stools (2), sore throat (1)
Enterovirus (*n =* 6)	2		4	
Respiratory syncytial virus (*n =* 3)				Nasopharyngeal secretions (3)
Rotavirus (*n =* 1)				Stools (1)
Clinical diagnosis (*n =* 15)				

*N*: number of infants with bacterial or viral infections.

**Table 2:** Demographic, clinical characteristics, and laboratory findings in infants with bacterial and viral infections on admission to the Unit and in controls.

	Bacterial infections (*N* = 19)	Viral infections (*N* = 28)	Controls (*N* = 18)
Gestational age (weeks)	38.5 ± 1.0	38.9 ± 1.0	38.6 ± 1.0
Birth weight (g)	3033 ± 285	3203 ± 379	3216 ± 406
Postnatal age (days)	17.4 ± 5.9	18.0 ± 6.6	17.0 ± 6.0
Males	13	16	11
Fever duration (hours)			
<8	12	19	
8–24	6	6	N/A
>24	1	3	
Max temperature (°C)	38.4 ± 0.56* (37.8–40.0)	38.2 ± 0.45* (37.8–39.5)	36.7 ± 0.08 (36.6–36.9)
Fever recession following admission (hours)			
<36	14	21	
36–72	5	4	N/A
>72	0	3	
WBC (/mm^3^)	16052 ± 6659^∗§^ (1970–25340)	10141 ± 4000 (3670–17560)	9227 ± 2630 (5260–14580)
ANC (/mm^3^)	8610 ± 4221^∗§^ (768–15176)	3816 ± 2533 (770–11388)	2867 ± 1257 (1052–5233)
Immature neutrophils (/mm^3^)	48.7 ± 172.0 (0–746)	0	0
Platelet count (/mm^3^)	505300 ± 200800 (37000–920000)	429000 ± 141000 (187000–806000)	448000 ± 171000 (250000–714000)
Urea (mg/dL)	26.5 ± 13.2^∗§^ (11.0–71.0)	14.1 ± 6.4 (4.0–30.0)	12.8 ± 7.3 (5.0–35.0)
Creatinine (mg/dL)	0.34 ± 0.14 (0.10–0.65)	0.29 ± 0.08 (0.18–0.48)	0.31 ± 0.18 (0.10–0.73)
SGOT (IU/L)	34.1 ± 25.7 (6.0–129.0)	37.2 ± 25.4 (20.0–148.0)	42.8 ± 12.9 (22.0–60.0)
SGPT (IU/L)	26.1 ± 8.8 (7.0–45.0)	26.6 ± 18.0 (9.0–104.0)	25.8 ± 14.2 (12.0–59.0)
*γ*-GT (IU/L)	87.6 ± 36.0 (30.0–163.0)	90.0 ± 43.0 (22.0–225.0)	110.8 ± 46.2 (41.0–207.0)
CRP (mg/L)	96.0 ± 81.2^∗§^ (0.2–273.0)	10.8 ± 28.9 (0.2–137.0)	0.56 ± 0.57 (0.2–1.8)
suPAR (ng/mL)	5.38 ± 1.92* (3.52–9.24)	4.73 ± 1.11^‡^ (2.69–7.83)	3.61 ± 0.74 (2.38–4.79)

N/A: not applicable.

Quantitative variables are expressed as mean ± S.D (range).

*P ≤ 0.001, ^‡^ *P* ≤ 0.05 compared with controls; ^§^ *P* ≤ 0.001 compared with the viral infection group.

## Abbreviations

suPAR: Soluble urokinase-type plasminogen activator receptor
CRP: C-reactive protein
WBC: White blood cell count
ANC: Absolute neutrophil count
SIRS: Systemic inflammatory response syndrome.
